# Comparison of Predictive Factors of Flu Vaccine Uptake Pre- and Post-COVID-19 Using the NIS-Teen Survey

**DOI:** 10.3390/vaccines12101164

**Published:** 2024-10-12

**Authors:** Ty J. Skyles, Harlan P. Stevens, Spencer C. Davis, Acelan M. Obray, Dashiell S. Miner, Matthew J. East, Tyler Davis, Haley Hoelzer, Stephen R. Piccolo, Jamie L. Jensen, Brian D. Poole

**Affiliations:** 1 Department of Microbiology and Molecular Biology, Brigham Young University, Provo, UT 84602, USA; ts569@student.byu.edu (T.J.S.); sdavis18@byu.edu (S.C.D.); east00@byu.edu (M.J.E.);; 2 Department of Biology, Brigham Young University, Provo, UT 84602, USAjamie.jensen@byu.edu (J.L.J.)

**Keywords:** influenza vaccination, COVID-19, demographic factors, machine learning

## Abstract

Background: Seasonal influenza vaccination rates are very low among teenagers. Objectives: We used publicly available data from the NIS-Teen annual national immunization survey to explore factors that influence the likelihood of a teen receiving their seasonal flu shot. Methods: Traditional stepwise multivariable regression was used in tandem with machine learning to determine the predictive factors in teen vaccine uptake. Results and Conclusions: Age was the largest predictor, with older teens being much less likely to be vaccinated than younger teens (97.48% compared to 41.71%, *p* < 0.0001). Provider participation in government programs such as Vaccines for Children and the state vaccine registry positively impacts vaccine uptake (*p* < 0.0001). Identifying as non-Hispanic Black was a small, negative predictor of teen vaccine uptake (78.18% unvaccinated compared to 73.78% of White teens, *p* < 0.0001). The state quartile for COVID-19 vaccine uptake also strongly predicted flu vaccine uptake, with the upper quartile of state COVID-19 vaccine uptake being significantly more likely to also get vaccinated for influenza (76.96%, 74.94%, 74.55%, and 72.97%, *p* < 0.0001). Other significant factors are the number of providers, education of the mother, poverty status, and having a mixed provider facility type. Additionally, the multivariable regression analysis revealed little difference in the predictive factors of vaccine uptake between pre- and post-pandemic datasets.

## 1. Introduction

Influenza viruses belong to the orthomyxoviridae family of viruses. These viruses are enveloped and have negative sense, single-stranded RNA genomes [1]. Influenza viruses mostly infect the lungs in humans but have been shown to affect other organs. Infection causes acute febrile illness with varying degrees of systemic and respiratory symptoms [2]. In addition to fever, symptoms of infection can include headache, chills, muscle weakness, sore throat, dry cough, and nasal discharge [3]. Most symptoms usually resolve within 3–5 days, but a cough and feelings of malaise have been shown to persist for up to two weeks [4].

Although influenza can infect people of any age, the highest instance of infection is in those who are under the age of 25 [1]. Infection of the elderly, immunocompromised, or those with coexisting chronic diseases can often cause life-threatening complications [2]. These viruses are fairly transmissible, with an R_0_ value between one and two [5]. Despite significant improvements in the prevention, control, and management of influenza infections, influenza has been endemic since the Middle Ages [6,7]. The worst influenza pandemic on record occurred in 1918, when the virus killed close to 50 million people worldwide [8]. Influenza’s rapid mutation rate is thought to be due to antigenic drift and shift, which the virus uses to mutate into new strains. These mutations commonly affect the hemagglutinin (HA) and neuraminidase (NA) genes, both of which play a role in entering and exiting cells [9].

Because influenza infections result in an average of 200,000 hospitalizations and 36,000 deaths each year in the United States, significant effort has been put into developing vaccines to prevent infection [3,4]. The efficacy of these vaccines varies from year to year, but they have been shown to be 33–67% successful in protecting individuals from infection [3,10,11]. The current recommendation by the Centers for Disease Control and Prevention (CDC) is that everyone over the age of 6 months receive the influenza vaccine each year. Despite this recommendation, only 57.8% of children 6 months to 17 years old were vaccinated during the 2021–2022 flu season, while only 49.4% of adults were vaccinated during the same time period. These percentages marked a decrease in vaccination coverage in both children and adults of 0.8% from the 2020–2021 flu season. Between the 2019–2020 and 2020–2021 flu seasons, child influenza vaccination rates decreased by 5.1% [5]. Research has shown that even low vaccination rates, especially among children and adolescents, can make a difference to the overall community disease burden [12,13].

Decreasing vaccine rates has largely been attributed to increasing rates of vaccine hesitancy, which is caused by various factors. These factors have been shown to include mistrust of authoritative institutions and healthcare, personal beliefs, customs, ideologies, and judgments around the risk versus benefit of vaccines [6,9,14,15,16]. Some researchers have studied the reasons behind influenza vaccine hesitancy using data from the National Immunization Survey-Teen (NIS-Teen), published each year by the CDC. The latest of these studies analyzed the immunization data between 2011 and 2017 [17,18]. Despite this, we found no studies since the COVID-19 pandemic of 2020 that compare the differences in pre- and post-pandemic influenza vaccination rates. Recognizing this, we analyzed the NIS-Teen survey data from 2016–2022 to better understand how the COVID-19 pandemic has influenced influenza vaccine hesitancy among teens in the United States.

## 2. Materials and Methods

This study used publicly available data from the Centers for Disease Control and Prevention (CDC) [https://www.cdc.gov/vaccines/imz-managers/nis/datasets-teen.html accessed on 20 May 2024]. Data were accessed during the period of 1 January 2024 to 1 May 2024. The authors had no access to potentially identifiable information, and consent was not obtained because the data were publicly available and not identifiable. We analyzed data gathered in the annual NIS-Teen nationwide survey, which is designed to gather vaccine data from a representative sample of the United States population. The NIS-Teen gathers data from both parents of teenagers and their healthcare providers. In the survey, random digit dialing is used to contact parents who have children aged 13–17. After completion of the telephone survey, the healthcare providers of the respondents were mailed a questionnaire to gather more information and to confirm the accuracy of the responses. Many households had more than one child. In these cases, one child was randomly selected for the survey [19]. The data used in this study have been made publicly available for research purposes and have gone through an extensive review by the National Center for Immunization and Respiratory Disease IRB [19].

Our analysis included NIS-Teen datasets from 2017, 2021, and 2022. To obtain a better understanding of what factors most impact flu vaccine uptake before and after the COVID-19 pandemic, the 2021 and 2022 datasets were combined and compared to the 2017 dataset. (The 2018 dataset was not used because it did not contain significant data about the flu vaccine.) After merging the datasets, they were cleaned. Only responses that reported the number of flu vaccinations a teen had received in the prior three years were included. Additionally, responses indicating that a teen had received more than three flu vaccines in three years were removed. Inconclusive responses such as “refused”, “unsure”, or “unanswered” were replaced with NA, but the entire survey responses were still included in the analysis.

The primary dependent variable in our study was the number of flu vaccines teens had received in the prior three years. The independent variables included diverse demographic factors (Table 1). These variables were chosen based on previous research on the NIS-Teen data [17,18,20]. The independent variable “State quartile” was created by ranking each state (and Washington D.C.) by COVID-19 vaccine uptake and then grouping them into quartiles. This was achieved using the method described by Leuchter et al. [20]. State quartile 4 represents the 13 states with the highest COVID-19 vaccine uptake, while state quartile 1 represents the 13 states with the lowest COVID-19 vaccine uptake.

Initially, contingency tables were made, and a chi-square analysis was performed. Regression analysis was performed on the 2021–2022 dataset. The number of flu vaccines received in the past three years was treated as a continuous variable, and all predictor variables were treated as nominal (except for age, which was ordinal). The dependent variable was analyzed as a continuous variable, and all independent variables were analyzed as nominal. The same multivariable regression was run using both the 2017 dataset and the 2021–2022 datasets. To compare the two, the regressions were not performed in a stepwise fashion. Instead, these regressions included all variables. To test the difference between the 2017 and 2021–2022 datasets, the datasets were recombined, and a variable was made that indicated if the response was from the 2017 or 2021–2022 dataset (cohort). We performed a regression that indicated the cohort had no impact on flu vaccine uptake (see Supplementary Materials S1), and we performed a regression in which we crossed each independent variable by cohort. All analyses were conducted using JMP Pro 16 (SAS Institute Inc., Cary, NC, USA, 1989–2023).

After the initial regression analysis, we created a machine-learning model to predict the number of flu vaccines teens had received in the past three years. The pipeline used to select, train, and optimize a model follows that used previously by Stevens et al. [21]. To build the model, we preprocessed both the 2017 and 2021–2022 datasets to only include the 17 variables chosen for the study: age of teen, poverty, education of parent, education of child, parent marital status, language, sex, asthma, race, number of valid providers, immunizations reported to registry, VFC order, well-child exam, age of parent, insurance status, and facility. Due to a low percentage of teens who had had two to three vaccines in the past three years, the dependent variable was converted into a binary variable. Any teen who had received one, two, or three vaccines in the past three years was encoded as one group, and any teen who had received zero vaccines in the past three years was encoded as a separate group. This was conducted because the number of teens who received two or three vaccines was too small to model as their own categories. Additionally, receiving at least one vaccine indicates that the vaccine decision maker is physically able to obtain a vaccine and is unlikely to be vaccine-hesitant. There is likely a small difference in demographics between those who received one, two, or three vaccines in three years, but that difference is unlikely to be measured with high confidence. Additionally, we converted all ordinal variables into integers and one-hot encoded all of the categorical variables [22]. After that, we split each dataset into a training set of 80% and a testing set of 20%. To avoid overfitting a model on the 2021–2022 dataset, model selection and parameter tuning were performed on the 2017 dataset. The best-performing model and parameters were then used in training a model and predicting vaccination uptake on the 2021–2022 dataset.

To determine which machine-learning model to use, we first evaluated a large number of machine-learning models without tuning the hyperparameters. After selecting the model that yielded the highest accuracy, we proceeded to optimize that particular model’s hyperparameters to maximize the area under the receiver operator curve (AUROC).

For model selection, we used the LazyClassifier function from the lazypredict (Shankar Rao Pandela, Bengaluru, India v0.2.10) package, which trains multiple machine-learning models from the sklearn (1.4.1) [23] package with default parameters on the testing set identified (20% of the 2017 respondents). Based on these calculated metrics (Supplementary Materials S2), we proceeded with XGBClassifier (https://arxiv.org/abs/1603.02754 accessed on 1 July 2024), as it had the highest F1 score (0.70) and accuracy (0.72) on the 2017 testing dataset. Although GaussianNB had a slightly higher AUROC score (0.68), the XGBClassifier has been extensively used in relevant literature [24,25,26]. XGBClassifier is the scikit-learn implementation of an extreme gradient-boosted (XGB) classification machine-learning model. XGB uses gradient boosting to iteratively combine multiple weak decision trees into a single predictive model [27].

We used Optuna (3.6.0) https://dl.acm.org/doi/10.1145/3292500.3330701 accessed on 1 July 2024) to optimize the hyperparameters for the XGBClassifier model on the 2017 dataset. From 100 trials and the AUROC as the maximization criteria, LazyClassifier suggested that the best parameters for the XGBClassifier model were as follows: number of boosting rounds = 452, maximum tree depth = 3, learning rate = 0.02627, subsample ratio = 0.7582, L1 regularization term = 6.7476, and L2 regularization term = 0.05761. Class 0 (respondents who received zero vaccines in the prior three years) was significantly more frequent than class 1 (those who received one or more vaccines in the last three years). Therefore, we assigned class weights that were inversely proportional to the class frequency.

Metrics for the model’s performance were calculated with sklearn (1.11.4) and included the AUROC, balanced accuracy, recall, precision, and the F1 score. The AUROC measures how well a model can discriminate between positive and negative classes across different thresholds for probabilistic predictions. Balanced accuracy indicates the proportion of correct predictions adjusted for class-label frequency. Recall, or the true positive rate, is the ratio of correctly predicted positive instances to the total true positive instances. Precision is the ratio of correctly predicted positive instances to the total predicted positive instances. The F1 score is a harmonic mean between precision and recall.

After training the XGBClassifier model on the 2021–2022 dataset with the parameters identified above, we used the *plot_importance* function from XGBoost to determine which features contributed to the model’s prediction. The “importance type” parameter used was ‘weight’, which estimates the feature’s importance by measuring the frequency of a feature that appears in a tree across the boosting rounds.

### Code and Data Availability

The processed NIS-Teen dataset used for machine learning can be found in Supplementary Materials S3. The final trained model, trained on a portion of the 2021–2022 dataset, can be found in Supplementary Materials S4. The code used to preprocess the NIS-Teen dataset, select and train a model, run predictions, and create figures is in Supplementary Materials S5.

## 3. Results

Between the 2017 and 2021–2022 surveys, a total of 55,342 respondents (20,549 from 2017 and 34,793 respondents from 2021–2022) were included in the study. Distributions of the responses from the 2021–2022 dataset can be found in Table 1. Demographics are thought to be generalizable to the teen population of the United States

Out of the 17 variables included in the study, the chi-square analysis showed a *p*-value of <0.0001 for 14 variables. A total of 41.71% percent of 13-year-olds had zero vaccinations compared to 97.48% of 17-year-olds, and 7.22% of 13-year-olds had three vaccines in the last three years compared to only 0.37% of 17-year-olds. The percentage of each state quartile with zero vaccines, from quartiles 1 to 4, was 76.96%, 74.94%, 75.55%, and 72.97%, respectively.

Teens were more likely to be vaccinated if their mother was a college graduate compared to if their mother was not a college graduate. Teens in the lowest state COVID-19 vaccine uptake quartile were less likely to be vaccinated against the flu than teens in the highest state COVID-19 vaccine uptake quartile. There was a large stratification in vaccination rates between age groups, with older teens being much less likely to be vaccinated than younger teens. Additionally, teens with no providers that order vaccines from VFC (Vaccines for Children, a program run by the CDC that provides free vaccines for children who are uninsured or underinsured) were less likely to be vaccinated.

**Figure 1:**
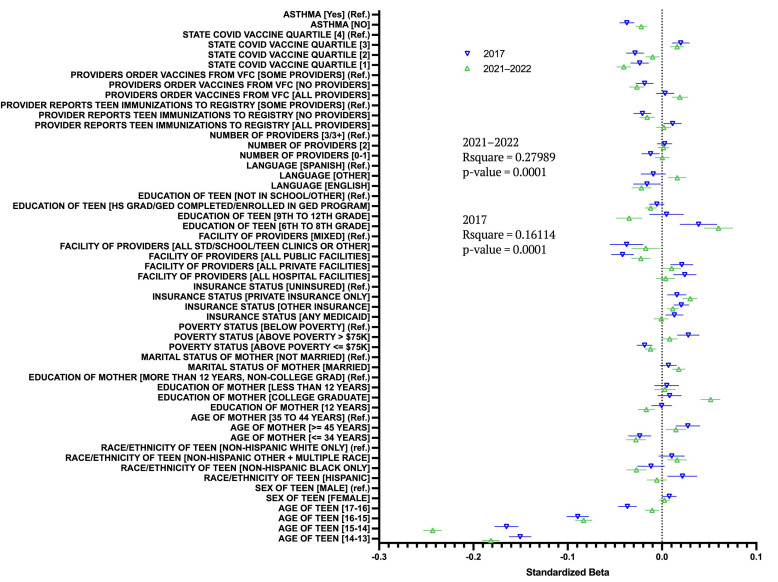
Regression analysis between different variables in a category for vaccination rates in 2017 and 2021–2022. Blue values show the results for the years 2017, while green values show the results for 2021–2022. The 2017 regression: F(38, 15,025) = 75.95, *p* < 0.001; 2021–2022 regression: F(38, 22,571) = 230.8649, *p* < 0.001. All statistical test results are located in Supplementary Materials S1.

To compare the predictors of flu vaccine uptake before and after the COVID-19 pandemic, regression analysis was performed on both the 2017 and 2021–2022 datasets. After crossing all variables by year, we compared the BIC (Bayesian information criterion) value for both models and observed that the BIC for the uncrossed model (80,236.2) was smaller than the crossed model (80,477.9) (Figure 1). We concluded that the cohort did not significantly change how any independent variable affected the likelihood of flu vaccine uptake. The results of this analysis can be found in Supplementary Materials S1.

An overarching motivation of our work was to evaluate the potential to predict flu vaccination status based on clinical and demographic variables. Such an ability could be useful in clinical settings to identify youth at the highest risk of not being vaccinated.

**Figure 2:**
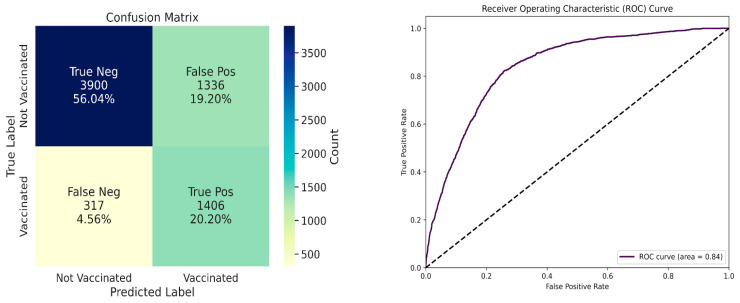
Receiver operating characteristic (ROC) curve and confusion matrix showing the performance of the XGBoost classification model on the test set to predict whether a given teen had been vaccinated in the prior three years. The area under the ROC curve (0.84) far exceeded the baseline expectation of 0.5.

Furthermore, it might be useful at a population level to inform public health decisions. For example, if we could estimate the vaccination rates among youth in a particular geographical area, it might provide insight into community disease burden and inform decisions about influenza vaccination campaigns. Because relatively few teens had received multiple vaccines in the prior three years, we converted the flu vaccination status to a binary variable, indicating whether individual youths had received at least one flu vaccine in the prior three years. We evaluated our ability to predict this status based on multivariate patterns among the independent variables. Since our non-stepwise regression analysis demonstrated that relationships between the independent variables and flu vaccination status were stable across years, we assumed that we could perform model selection and identify the optimal hyperparameters for a classification model by using the 2017 data only and that these findings would generalize to the 2021–2022 data. This process identified XGBoost as the best-performing algorithm (see Methods). We then trained this algorithm on data from 27,834 respondents from the 2021–2022 data and made probabilistic predictions for the remaining 6959 respondents (test set). The predictions resulted in an AUROC of 0.84 (Figure 2). Table 2 and Figure 2 provide additional insight into the model’s performance based on a probability threshold of 0.5. Of the test-set respondents, 5236 (75.2%) reported not having received the flu vaccine in the prior three years; the model correctly identified 3885 (74.3%) of these respondents. Of the 1723 test-set respondents who had not received the flu vaccine in the prior 3 years, the model correctly identified 1406 (81.6%) of these individuals. Figure 2 illustrates the class imbalance (far more unvaccinated teens than vaccinated) and its tendency to over-predict the frequency of teens who are vaccinated. Accordingly, it appears our efforts to adjust for the imbalance between vaccinated and unvaccinated individuals were helpful.

**Figure 3:**
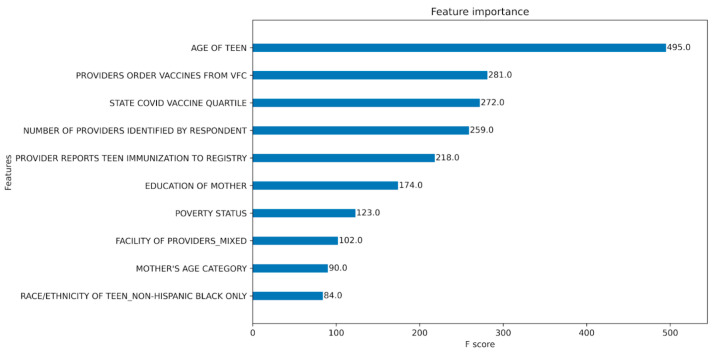
Predictive power of the top ten variable/sub-variables in the XGBoost classification model. The F score indicates the frequency with which a given independent variable is present across the decision trees used by XGBoost.

To determine which features were most informative for the XGBoost model, we quantified the frequency with which a given independent variable appeared in a decision tree across the boosting rounds (Figure 3).

## 4. Discussion

Both traditional statistical methods and machine learning indicated that age was by far the most important predictive factor for flu vaccine uptake. Previous research has shown that teens visit the doctor less often as they get older, and it is thought that this provides fewer convenient opportunities to become vaccinated [28]. Although older teens are not an especially disease-vulnerable group, their contact networks are highly heterogeneous, and teenage social groups are thought to be centers of disease transmission [29]. Increased vaccination rates among this group could have a large impact on the spread of seasonal influenza and other diseases. Providing convenient opportunities for teens to be vaccinated, such as at school, could lessen the prevalence and disease burden of seasonal influenza significantly.

Our data add to a growing body of literature that supports the theory that vaccine hesitancy and vaccine uptake patterns are similar across multiple different vaccines [8,20]. It is thought that the same attitudes and barriers that influence COVID-19 vaccine decisions also influence flu vaccine decisions. This is becoming increasingly relevant as COVID-19 has begun to exhibit seasonal infection patterns [30]. We anticipate that as COVID-19 boosters become similar to seasonal flu vaccines, vaccine-hesitant people will treat the two similarly. Public health messaging and vaccination campaigns will likely have to address patterns in COVID-19 vaccine uptake that will bleed into flu vaccine uptake and vice-versa.

Two significant predictors of flu vaccine uptake were whether or not a teen’s provider(s) ordered vaccines from Vaccines for Children and whether or not a teen’s provider(s) submitted data to the state vaccine registry. The data show that a teen is more likely to receive their flu vaccine if their provider participates in the VFC program. This is a reflection of the success and continued importance of the program, as well as the importance of primary care providers in pediatric vaccine uptake. A large body of research supports the importance of provider recommendation in flu vaccine uptake within most demographics [31,32,33]. The impact of ordering vaccines from VFC on teen flu vaccine uptake can be possibly explained in two ways. First, providers who order vaccines from the VFC program are inherently more concerned with the vaccination status of their patients and are thus more likely to promote vaccination in their clinic, and second, patients are more likely to become vaccinated when the clinic provides an opportunity for vaccination at little to no cost. Both explanations are likely true. Additionally, participating in the state vaccine registry likely reflects a provider’s concern for the vaccination of their patients. The impact of ordering from VFC and participating in the vaccine registry highlights the importance of primary care providers in vaccine uptake.

Increased education of mothers appears to be a significant predictor of flu vaccine uptake. It is thought that education provides an understanding of basic sciences that decreases vaccine hesitancy, but there is a possibility for potential confounding variables. Previous research supports a strong correlation between vaccination and maternal education in the developing world and a weaker correlation in the developed world [34]. More research is needed to explore the relationship between maternal education and child vaccine uptake in developed countries.

Our model shows that identifying as being non-Hispanic Black was a negative predictor of flu vaccination. The effect is small compared to other factors, but it is consistent with previous research in the field [35,36]. The differences in non-vaccination rates between non-Hispanic Blacks (78.18%) and non-Hispanic Whites (73.78%) are thought to be moderated by a complex array of cultural and socioeconomic factors [37]. A useful evaluation of these factors is not possible using NIS-Teen survey data.

Our data shown in Figure 2 indicate that the demographic factors in 2017 predict flu vaccine uptake in similar ways to 2021–2022. Although the current research suggests that the COVID-19 pandemic has had an impact on flu vaccine uptake [8,20], our data suggest that the demographics of those who are vaccine-hesitant have not changed significantly over the course of the pandemic. The same groups of people who were unlikely to be vaccinated in 2017 were still unlikely to seek vaccination in 2021–2022.

Vaccination rates have also been seen to be low in older teens and young adults in a university setting [38]. In adults, influenza vaccination tends to be higher among women and those with commercial insurance. Interestingly, those in higher-poverty areas tended to be more likely to be vaccinated [39]. This is in line with our findings that patients of providers who participate in vaccine programs are more likely to be vaccinated.

## 5. Conclusions

The United States population of teens continues to have a low flu vaccination rate. Teen flu vaccine uptake is influenced by the teens, their parents, and their providers. Future vaccine initiatives should target older teens and provide them with free and convenient opportunities to be vaccinated. Providers have a large impact on flu vaccine uptake. Programs that seek to improve teen vaccine uptake could be more effective by providing training and low-cost vaccines to primary care providers. Vaccine hesitancy towards the flu vaccine is expected to follow similar trends as vaccine hesitancy towards the COVID-19 vaccine, especially as COVID-19 is beginning to show seasonal trends. Race continues to be a small but significant predictor of vaccine uptake. Flu vaccine uptake is subject to many social, cultural, economic, and logistical barriers that should be addressed while trying to decrease the flu burden for all in the community.

## Figures and Tables

**Table 1:** Demographic information and descriptive statistics stratified by number of flu vaccinations in the last three years. A total of 14 out of the 17 variables included in the study had a chi-square *p*-value of <0.0001. We included 34,793 people from the 2021–2022 NIS-Teen survey. Note: not all variables will add up to 34,793 due to incomplete responses.

			Flu Vaccinations in Past Three Years (%)				
Variable	Category	Number of Respondents	0	1	2	3	*p*-Value
Total Vaccines Received n = 34,793			26,029	5461	2618	685	
Asthma	Yes	6802	74.3	16.33	7.37	2	0.4106
	No	27,939	74.93	15.53	7.57	1.96	
Age of Teen	13	7027	41.71	27.14	23.94	7.22	<0.0001
	14	7321	55.92	32.93	10.01	1.13	
	15	7051	85.52	12.48	1.49	0.51	
	16	6914	96.28	2.43	0.78	0.51	
	17	6480	97.48	1.47	0.68	0.37	
Poverty Status	<=USD 75 k	9639	77.39	15.04	5.97	1.6	<0.0001
	>USD 75 k	19,644	72.78	16.16	8.82	2.24	
	Below poverty	4465	77.81	15.05	5.69	1.46	
Education of Mother	12 years	5084	78.48	14.36	5.59	1.57	<0.0001
	College graduate	18,834	72.25	16.56	8.85	2.35	
	<12 years	2064	76.89	15.16	6.49	1.45	
	>12 years, non-college graduate	8811	77.69	14.75	6.05	1.51	
Education of Teen	6th–8th grade	9518	45.43	27.67	21.23	5.66	<0.0001
	9th–12th grade	24,766	85.72	11.31	2.39	0.58	
	HS grad/GED Completed/enrolled in GED program	322	98.76	1.24	0	0	
	Not in school/other	144	80.56	13.89	3.47	2.08	
Marital Status of Mother	Married	23,957	73.49	16.02	8.3	2.2	<0.0001
	Never married/previously Married	10,836	77.74	15.7	7.52	1.97	
Language	English	32,450	74.41	15.82	7.73	2.03	<0.0001
	Other	224	69.64	18.3	9.82	2.23	
	Spanish	2119	81.55	13.45	4.06	0.94	
Race/Ethnicity of Teen	Hispanic	6608	77.07	15.63	5.75	1.54	<0.0001
	Non-Hispanic, Black only	3258	78.18	14.36	6.05	1.41	
	Non-Hispanic Other + Multiple Race	4223	73.72	16.5	7.86	1.92	
	Non-Hispanic White only	20,704	73.78	15.76	8.25	2.2	
Sex of Teen	Female	16,433	74.52	15.83	7.63	2.01	0.6762
	Male	18,360	75.07	15.57	7.43	1.93	
Number of Providers	0–1	16,902	75.66	15.25	7.25	1.85	<0.0001
	2	10,833	74.19	15.99	7.82	2	
	3/3+	7058	73.73	16.32	7.74	2.21	
Provider reports immunizations to registry	All providers	20,923	73.91	16.09	7.84	2.16	<0.0001
	No providers	1587	77.95	13.3	6.74	2.02	
	Some, but possibly or definitely Not all providers	5093	70.12	18.18	8.99	2.71	
Providers order vaccines from VFC?	All providers	19,134	73.95	15.86	7.94	2.24	<0.0001
	No providers	2686	78.33	13.03	6.85	1.79	
	Some, but possibly or definitely Not all providers	5819	69.91	18.92	8.68	2.49	
State COVID vaccine quartile	1	7367	76.96	15.41	6.49	1.14	0.0003
	2	7975	74.94	15.21	6.57	1.28	
	3	9053	74.55	16	7.77	1.68	
	4	9379	72.97	17.1	8.02	1.91	
Mother’s Age Category	<=34 Years	2105	67.27	21.71	8.55	2.47	<0.0001
	>=45 Years	17,920	77.53	14.05	6.82	1.6	
	35–44 Years	14,768	72.58	16.83	8.23	2.35	
Insurance Status	Any medicaid	10,401	76.98	15.3	6.14	1.58	<0.0001
	Other insurance	2573	74.58	15.93	7.77	1.71	
	Private insurance only	20,700	73.23	16.12	8.39	2.26	
	Uninsured	771	83.66	10.64	4.67	1.04	
Facility of Provider	All hospital	3995	74.47	15.97	7.73	1.83	<0.0001
	All private	13,005	74.08	15.5	8.04	2.38	
	All public	3360	78.69	14.08	5.65	1.58	
	All STD/school/teen clinics/other	991	79.92	13.32	5.85	0.91	
	Mixed	7230	70.09	15.93	7.79	2.19	

**Table 2:** Classification metrics for XGBoost predictions on the test set. A positive class label indicated that a respondent had received a flu vaccine in the prior three years.

Metrics	Value
Area Under ROC curve	0.84
Balanced Accuracy	78.04%
Precision	0.51
Recall	0.82
F1 Score	0.63

## Data Availability

Data were obtained from publicly available sources and are available here: https://www.cdc.gov/vaccines/imz-managers/nis/datasets-teen.html, accessed on 5 May 2024.

## Supplementary Materials

The following supporting information can be downloaded at: https://www.mdpi.com/article/10.3390/vaccines12101164/s1, Datasets S1, S2, S3, S4, and S5.
